# MindSeer: a portable and extensible tool for visualization of structural and functional neuroimaging data

**DOI:** 10.1186/1471-2105-8-389

**Published:** 2007-10-15

**Authors:** Eider B Moore, Andrew V Poliakov, Peter Lincoln, James F Brinkley

**Affiliations:** 1 Structural Informatics Group, Departments of Biological Structure and Medical Education and Biomedical Informatics, University of Washington, Seattle, USA; 2 Department of Computer Science and Engineering, University of Washington, Seattle, USA

## Abstract

**Background:**

Three-dimensional (3-D) visualization of multimodality neuroimaging data provides a powerful technique for viewing the relationship between structure and function. A number of applications are available that include some aspect of 3-D visualization, including both free and commercial products. These applications range from highly specific programs for a single modality, to general purpose toolkits that include many image processing functions in addition to visualization. However, few if any of these combine both stand-alone and remote multi-modality visualization in an open source, portable and extensible tool that is easy to install and use, yet can be included as a component of a larger information system.

**Results:**

We have developed a new open source multimodality 3-D visualization application, called MindSeer, that has these features: integrated and interactive 3-D volume and surface visualization, Java and Java3D for true cross-platform portability, one-click installation and startup, integrated data management to help organize large studies, extensibility through plugins, transparent remote visualization, and the ability to be integrated into larger information management systems. We describe the design and implementation of the system, as well as several case studies that demonstrate its utility. These case studies are available as tutorials or demos on the associated website: .

**Conclusion:**

MindSeer provides a powerful visualization tool for multimodality neuroimaging data. Its architecture and unique features also allow it to be extended into other visualization domains within biomedicine.

## Background

The problem we address in this report is visualization of integrated multimodality three-dimensional (3-D) brain imaging data. Such visualizations can lead to new insights that are not apparent from a single modality alone.

Over the past several years many applications have become available that include some aspect of 3-D visualization. A comprehensive listing of these applications can be found by searching the Harvard Internet Analysis Tools Registry (IATR) [1] for those tools that are indexed by the keyword "visualization". As of March 2007 such a search returned a list of 116 tools. Of these tools many are designed for analysis of specific types of data; as, for example, LORETA for source localized EEG [2]; FSL [3], SPM [4] and AFNI [5] for functional magnetic resonance (fMRI) data; and JAtlasView [6] and the Allen Brain Atlas [7] for gene expression data. Because they are designed for only one type of data these tools generally do not display different types of data at once.

On the other end of the spectrum, many tools are general purpose, such as Analyze [8], 3DVienix [9], VTK [10] or VolView [11]. These tools can integrate and visualize many different kinds of data. However, if a user is simply interested in visualization of existing integrated data they can be overkill, especially given that some of them are expensive. Somewhere in the middle are programs specifically designed for multi-modality 3-D biomedical data, such as MRICro [12], MedX [13], BrainSuite [14], LONI Visualization Environment [15], FreeSurfer [16], Caret [17], NeuroTerrain [18], and 3D Slicer [19]. However, because many of these programs contain extensive analysis or other methods they may be more complex than is needed for pure visualization. In addition, few if any of these programs combine both stand-alone and remote multi-modality visualization in an open source, portable and extensible tool that is easy to install and use, yet can be included as a component of a larger information system.

In this report we describe such a 3-D visualization program, called MindSeer, which combines many of the best features of existing 3-D brain visualization programs, yet is greatly simplified by restricting it to visualization of existing data. The program is written in Java and Java3D for true portability, and includes integrated data management, interoperability with many common neuroimaging data formats, standalone operation as well as remote visualization of centralized data through client-server access, the capability to be a component in a larger information system, and extensibility through plugins. The program is freely available, portable across all three major platforms (Windows, Mac, Linux), and runs at interactive speeds when installed locally.

The remainder of this report describes the design goals and implementation for MindSeer, and as well as examples of its use. More extensive documentation, downloads, and tutorials are available on the project web page [20].

## Implementation

### Design Goals

We have designed MindSeer to make visualizing and managing complex multi-modality neuroimaging datasets easy and convenient. In order to provide the most intuitive system possible, we have kept MindSeer focused on visualization, avoiding the complexity of duplicating analysis tools that are already found within existing programs. We have therefore designed MindSeer to work with the output of several popular brain image analysis tools, such as SPM [4] and FSL [3], while providing a plugin mechanism for importing and exporting data from other analysis packages. Table 1 lists the current compatible file formats.

**Table 1:** Supported file formats

NIFTI
Analyze 7.5 (with SPM's extension)
MINC
PNG, JPEG, GIF, TIFF (with download from Sun)
Matlab file containing a surface
GeomView
XML based map file

Within this focus on visualization, we have defined a set of design goals. MindSeer should:

1. Import and export common data formats.

2. Provide both 2-D slice views and 3-D surface views with cutaways.

3. Allow superposition of multiple sources and types of functional data on a single structural volume or surface.

4. Be easy to setup, with no configuration for the end user.

5. Be easy to extend. New neuroimaging data sources and visualization techniques are in continuous development.

6. Support integrated data management, and provide a system where it is easy to keep track of images and visualize them in new and different scenes.

7. Support these functions and others in a client-server mode that transparently provides interactive visualization of data in a central repository.

8. Be portable across all major platforms, and run at interactive speeds.

9. Be able to be a component in a larger information system.

10. Be freely available under an open source (GPL) license.

### Architecture

The architecture of MindSeer is designed to meet these goals. The program is written in Java and Java3D [21], so is platform independent. Recent versions of Java3D take advantage of the considerable rendering speeds of desktop graphics cards, so are able to provide real-time rendering of large 3-D surface models, yet retain portability.

**Figure 1:**
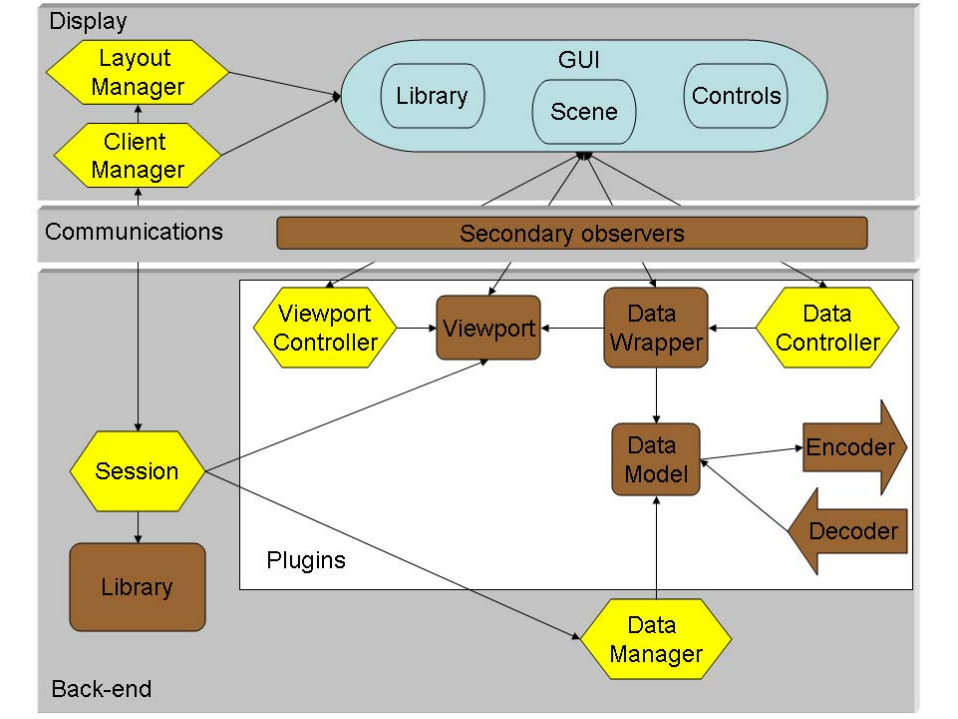
**System Architecture**. An overview of the general architecture of MindSeer, showing the main classes. The system follows the Model-View-Controller design pattern, and is divided into 3 main parts (Display, Communications and Back-end). Yellow classes are controllers, brown are models, and blue are views. The white box in the Back-end encloses plugins: abstract classes that are subclassed for specific data types and visualization methods.

To meet the need for transparent client-server mode with the same look and feel as the standalone version, MindSeer is designed in 3 main parts based on the model-view-controller (MVC) pattern [22]. The MVC pattern separates the user interface (View) from the underlying data representation and processing (Model) by introducing an intermediary (Controller) to respond to input and changes. In our case, the display and the communication layer form the view, and the back end contains both models and controllers (Figure 1). This division is natural because MindSeer can either work as a standalone application where the display and back end communicate instantaneously, or can be separated, with the display becoming the client application, the back end becoming the server, and the communication layer taking care of synchronization.

The display is further divided into a number of subparts (Figure 1, top). The main work horse is the Client Manager. This component acts as an intermediary to ensure that communication between the display and back end is executed in a deterministic order in a multi-threaded environment. It also monitors the scenes that are currently visible, and manages events to load new scenes and add data to existing scenes. Another display controller is the Layout Manager, which takes care of laying out the components of the Graphical User Interface (GUI). The Layout Manager can be changed to support different layouts; currently the system supports both a single tabbed window layout and a layout with multiple floating windows. The third important piece is the GUI, which consists of the displayed scene, widgets for controlling the data and scene, and the library view, which displays the available files of neuroimaging data and associated metadata.

Within the GUI communications are primarily handled by a special set of GUI widgets that are based on the observer pattern [23]. This pattern allows an object, the observer, to register itself with the subject. The subject will then notify all registered observers whenever important changes or events occur. The widget itself is the subject and the primary observer is the controller on the back-end. For example, in Figure 1 the Viewport Controller is an observer for the view control widgets in the GUI. When in client-server mode, secondary observers act as the communication link between the two computers by transmitting user events and view changes across the network connection. In the few cases where communication is not graphically initiated, such as opening a session, quitting or launching a new view, the client manager will send an event to the session on the back-end.

The back end (Figure 1, bottom) provides an extensible framework for processing data and generating the content of scenes. The framework is directed by a Session object, which controls the flow of the program. The session is aided by a Data Manager, which loads and caches the neuroimaging data files in a memory efficient manner. The master copy of the Library is maintained by the Session. Scenes are also maintained and created in the Viewport by the Session.

### Plugins for managing and viewing scenes

New data types and methods for displaying them in scenes are added to the system by subclassing one or more of seven abstract plugin classes (box in the lower part of Figure 1), which provide a set of common methods for each plugin type. The seven classes all interrelate, and include file Decoders and Encoders, Data Models, Data Wrappers, Viewports, View Controllers and Data Controllers. The Decoders and Encoders are responsible for loading a file and turning it into a compatible Data Model. The Data Model represents a shared form that the file takes in memory, whereas Data Wrappers present a Data Model in the form required by a specific type of Viewport. Viewports are responsible for rendering a scene using wrapped versions of each Data Model. Viewports also support custom controls and algorithms through Viewport Controllers and Data Controllers, which can be customized for each type of Data Wrapper and Viewport.

The specific instances of these seven abstract classes are discovered at runtime by consulting an XML configuration file that describes where to find these concrete implementations. The concrete implementations are registered with factory classes that produce the correct concrete implementation from information known at runtime. For example, the decoder for a NIFTI [24] file can be retrieved by simply asking the factory for a NIFTI decoder. Likewise, the appropriate default viewport can be called just by telling the factory what data model is being used.

The base system provides two main sets of prebuilt plugins for visualizing volumetric datasets (i.e. MRI or source localized EEG) and surfaces (i.e. the cortical surface). These plugin sets are organized around a slice viewport and a surface viewport.

The slice viewport displays volumetric data in coronal, transverse and sagittal views. It is capable of layering an arbitrary number of volumes and coordinate maps. To aid in differentiating the volumes and maps, each can be assigned its own color scheme and transparency value. This viewport is supported by several plugins, including decoders for NIFTI, Analyze 7.5, SPM's version of Analyze 7.5, and a custom XML map file of 3-D coordinates within or on the surface of the brain (Table 1). The decoders can produce two different kinds of data models: the volume data model, which represents 3-D and 4-D (time series) volumes such as MRI and source localized EEG, and the map data model, which represents the XML map file. These models are in turn encapsulated by data wrappers for different types of visualization.

Three dimensional surfaces are displayed in the surface viewport, which supports dynamic interaction and transparency, and can display any number of surfaces. Surfaces can either be pre-generated using a tool such as SPM, or isosurfaces can be generated by MindSeer from the volumetric data. Like the slice viewport, the surface viewport is supported by several plugins. This viewport has its own decoders for SPM's 3-D surface format and some of the GeomView [25] surface file formats, but it also shares the decoders for volume and map files with the slice viewport. A surface data model handles surface data files, and data wrappers over the surface data model hold display and color information in addition to producing the required Java3D objects.

By writing different wrappers for the surface and volume data models the same models can be re-used in both the volume and surface viewports. For example, there are three different wrappers for volume data models that produce three different visualization effects in the surface viewport: volumes as isosurfaces, volumes as orthogonal planes that cut into 3-D surfaces, and volumes as highlights over 3-D surfaces.

In addition to the two main viewports there are two minor viewports: an independent display of map file 3-D coordinates in tabular form, and a simple image viewport that displays digital photographs and other images.

To visualize a new data model or create a new way of visualizing an existing data model within an existing viewport, developers can write a new data wrapper to make the modality compatible with the viewport. In addition, developers can expand the variety of viewports beyond the slice and 3-D surface viewports.

New file types can be added by developing decoders and encoders to read and write them. To aid the development of new file decoders, MindSeer includes many tools and algorithms for manipulating and displaying data that is represented in typical ways. For surface data, the framework includes tools for handling and optimizing triangle arrays (both indexed and unindexed), which are a standard way to represent polygonal structures. For volumetric images, the basic built-in model is a 5 dimensional array that aligns closely with the NIFTI format and can accommodate most volumetric modalities and their file formats (image stacks, MINC, Analyze, etc.). The available data structures also support associating labels with individual intensities in a volume file. This gives MindSeer's built in volume model the capability to be extended to handle the custom lookup files that are associated with indexed atlases (e.g. the SHIVA atlas format [26]). These core data models – and the fact that they are decoupled from the details of file encoding and decoding – should allow MindSeer to model most medical imaging data.

### Data Management

**Figure 2:**
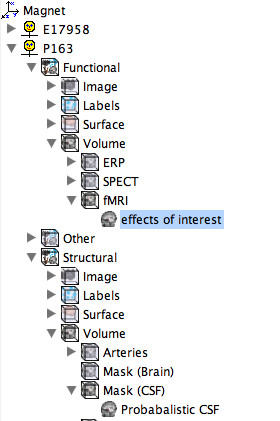
**MindSeer Library**. A screen capture showing the tree form of the MindSeer Library, where the Library is specified by an XML file defining the input/output file specifications for a given visualization. This particular library is organized by coordinate space (Magnet), subject (P163), type (Functional, Structural), file type (Image, Surface, Volume) and modality (fMRI, EEG). All of these metadata are internally stored in free form XML tags.

An important feature of MindSeer is that it integrates data management with visualization. By combining these functions, MindSeer provides a quick and easy way to create and experiment with scenes, and to automatically find data without user intervention. The application manages data in an XML file, called the Library, which contains file locations and associated metadata. Data can be tagged with any metadata, but the interface makes it easy to attach certain core tags, including the coordinate space (magnet, Talairach, etc.), the subject or patient identifier, whether the image is structural or functional, and the modality (MRI, fMRI, etc.). These core tags are used to automatically generate a consistent tree for easily browsing the data (Figure 2), which is sorted by coordinate space, subject, function versus structure, and modality. The system also infers the tags of a new data file based on where it is inserted into the tree. The core tags and exact tree structure are specified in an XML based template file that can be edited for other domains. Simple scripts can be created to generate the XML library file automatically from a database.

### Client-Server

By designing the system with separate display and back ends a client-server mode is a natural extension. MindSeer only required minor modifications and additions to add client-server functionality. For example, the server required the addition of a control class to generate new sessions for each client. This class also registers itself with the Java RMI [27] server so that clients can find it. Other additions include the introduction of secondary observers to automatically handle synchronization when either the client or server state changes. These observers are automatically added to observable subjects when the subjects are marshaled to be sent over the wire. Through this mechanism, we can create custom objects that transparently handle client-server mode. In addition the system tries to maintain interactivity over a variety of connections by adjusting image quality and compression level to reflect the network speed.

### GUI Widgets

The key to making it easy to write plugins that work in both standalone and client-server modes is that most communication is handled transparently through custom GUI widgets. These widgets are based on the architecture of Java Swing [28], and they therefore follow modified model-view-controller and observer patterns. While each widget can have any number of observers, in MindSeer observers usually are of two types. The first types are controllers, which handle events from the user and initiate changes in the scene. The second types are the synchronization listeners that are automatically added as the widgets are marshaled. Each synchronization listener monitors a copy of the widget for any property changes, and when they occur, the changes are propagated across the network. Additionally, in order to leverage the Java library, the widgets generate Swing objects for display.

### User Interface

**Figure 3:**
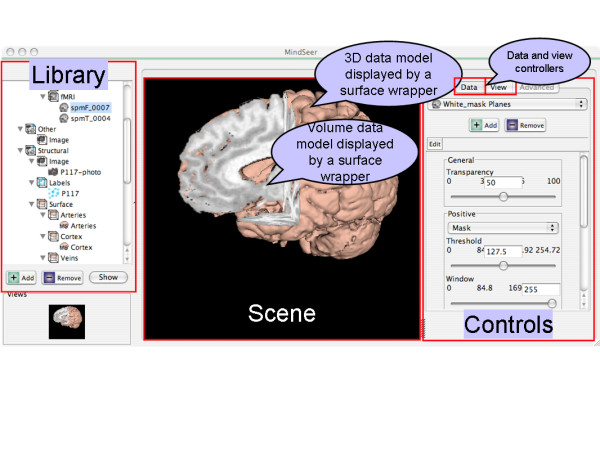
**User Interface**. This screen capture shows the default layout of MindSeer with the Library, Scene and Controls. It also shows how the plugins work together to generate a scene.

The user interface is designed to maximize consistency. Everything has a well-defined place (Figure 3), corresponding to the GUI classes in Figure 1. The viewport containing the scene is flanked by the Library view on the left and the scene Controls on the right, which are in turn divided into two tabs: Data and View. The Data tab contains controls for the appearance of an individual file within a scene. These controls can affect color, transparency, location, or other attributes. Additionally, since some files can be edited, this group of controls can be locked or unlocked by activating an "Edit" mode. The View tab, on the other hand, controls the appearance of the whole scene, including the order that volumes are stacked (influencing transparency), the camera parameters, and the location and extent of a wedge cut out of a surface.

The library view (as defined by the XML library file, left-hand side of Figure 3) is also kept visible and is intuitively interactive. The tree supports drag-and-drop for rearranging the library and transparently altering metadata, as well as drag-and-drop for adding files to existing scenes. To maximize user friendliness, MindSeer also provides several standard menus where preferences can be adjusted and changes undone.

## Results

In this section we demonstrate the range of MindSeer features by presenting a number of example case studies. Tutorials or demos of several of these use cases are presented on the website [20].

### Visualizing MRI, fMRI and source localized EEG

The focus of the system from the beginning has been to build rich scenes with diverse sources of data. Such data integration provides a qualitative feel for how different functional modalities interrelate and how they relate to the underlying structure. In order to test this capability, we obtained image volumes from three different imaging modalities: structural MRI, fMRI, and source localized EEG. We then displayed these volumes in a set of complex scenes. From the structural MRI we also derived the cortical surface and masks of the brain.

**Figure 4:**
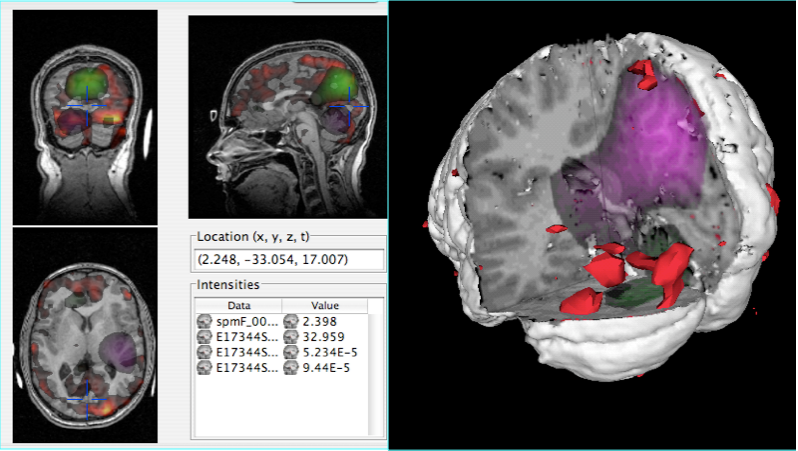
**Visualization**. Left. Functional and structural volume data shown in the Slice Viewport, which displays three orthogonal slices. fMRI (warm colors), and 2 related source localized EEG volumes (green and magenta) are simultaneously shown superimposed on the structural MRI. Right. The same data shown in the Surface viewport. In this case the fMRI is shown as red iso-surfaces and the EEG data are shown with a cutaway view.

The initial step was to build a library that had all the files, which was done by using the file menu to define a new library based on a template, and to add new image files in their proper location in the template. After placing each file into the library, we built a 2-D or 3-D scene by dragging an icon representing each desired data file from the library into a 2-D or 3-D viewport. The first such scene is a simple slice view with the structural MRI as a base and the fMRI and EEG each shown in different colors (Figure 4, left). The second scene is in 3-D. The scene starts on a base of the cortical surface. When we added the first functional volume to the surface the system used the library to automatically add the structural MRI and masks as it cut out the wedge. To achieve the final image, we generated 3-D isosurfaces of the functional data in order to pinpoint activation (Figure 4, right).

This entire scene was generated quickly and painlessly. Since MindSeer is compatible with tools researchers already use for analysis and segmentation, the addition of multimodality visualization is not an undue burden.

### Plotting 3-D Points from a 2-D Photograph

**Figure 5:**
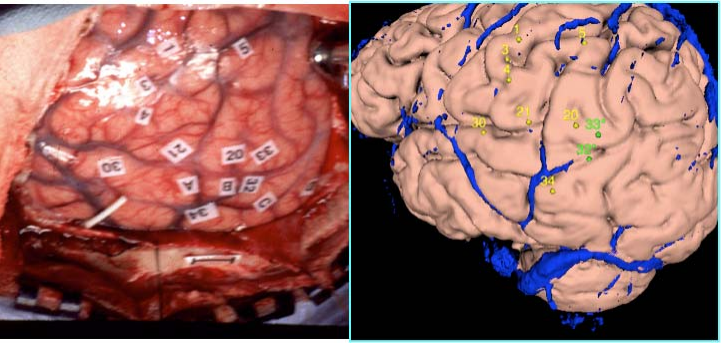
**Plotting Coordinates**. Left. A surgical photograph with Cortical Stimulation Mapping (CSM) sites. Right. A cortical surface of the patient's own brain onto which we have plotted the CSM points. Veins are in blue, and provide landmarks that enable the user to more easily match the photograph with the surface than would be possible with sulci and gyri alone. Sites that have been found to be significant for language are colored green.

Another feature of the system allows the user to indicate the 3-D locations of cortical landmarks on a displayed brain surface, in the process generating the XML map file described earlier. In our own work we use this feature to visually (i.e. by "eye") map the locations of labels from a surgical photograph (Figure 5, left) onto a 3-D surface rendering of the patient's own brain (Figure 5, right) [29]. The labels on the photograph show the locations where areas of exposed cortex were electrically simulated during a procedure called Cortical Stimulation Mapping (CSM) [30], which is used during neurosurgery to map the locations of language-sensitive cortex. By mapping these CSM sites to the patient's own brain and later to a normalized brain atlas we can relate them to other brain mapping data such as fMRI or single unit recording (SUR) [31].

The first step in this procedure was to use SPM to create a mask volume that strips away the skull from an MRI structural image volume, and to use the mask to generate a 3-D cortical surface. Given these data files (saved in Analyze format by SPM) we created a MindSeer Library pointing to these files, as well as the surgical photograph (Figure 5, left), the structural MRI image volume, and an MRI venogram showing cortical veins. The veins, in conjunction with sulci and gyri, are useful landmarks for the visual mapping procedure. Within MindSeer we then used the venogram and the mask generated in the first step to create a 3-D surface reconstruction of the patient's veins. This surface was added to a MindSeer scene showing the cortex and veins, which was displayed alongside the surgical photograph. The final step was to use the mouse to indicate the locations on the 3-D surface that correspond to the CSM sites shown on the photograph (Figure 5, right). The resulting dataset was then saved as an XML map file.

This overall plotting process is similar to our earlier Visual Brain Mapper [29], but has MindSeer's advantages of portability, remote access, and integration with analysis applications.

### Remote Visualization

An important concern for us from the beginning has been remote visualization. Over several years of research we have accumulated a repository and associated database with MRI, CSM and fMRI data from over 70 patients. Our collaborators need to be able to visualize this plethora of data without the added effort of downloading or organizing hundreds of gigabytes of files. To accomplish this, we harness the client-server mode of MindSeer. In this case the server accesses an XML Library that is automatically generated from the output of our X-Batch program [32]. X-Batch is a plugin for SPM that transparently generates a database describing (among other things) the locations of the structural and functional image files, accumulating multiple runs in a single file that is periodically converted to the MindSeer XML library format. Thus, the central repository is automatically updated whenever new MRI images are processed, thereby making these images immediately available for remote viewing in MindSeer. A demo of this capability for a sample dataset can be found on the project website [20].

When run in client-server mode the client-side user interface looks exactly like the interface for the standalone version. The only difference is in frame rates. While the client-server version lacks the fluidity of the standalone version, it is still quite interactive (Table 2), with frame rates of around 5 per second over a cable modem (200 milliseconds per frame).

**Table 2:** MindSeer performance on a number of setups and networks

**Configuration**	**Rendering time for complex 3-D scene (milliseconds)**
Standalone – modern hardware with integrated graphics card	50
Standalone – modern hardware with dedicated graphics card	10
Client-server – Cable modem	200
Client-server – Local connection over 100 mb network	50

### Integration within a larger information system

As part of our Human Brain Project [33] work our group has created several local Laboratory Information Management Systems to manage various types of multi-modality brain imaging data. These databases include the X-Batch XML database (described in the last section) for managing MRI data processed by SPM [32], as well as separate relational databases for cortical stimulation mapping (CSM) [34] and single unit recording (SUR) [35] data. These separate databases are encapsulated in web services that accept queries in the XML query language XQuery [36-38]. A distributed XQuery processor [39] accepts queries over one or more of these data sources, sends individual query fragments to the separate data sources, and packages the separate results into a single XML file. A web-based application called DXBrain [40] allows the user to formulate distributed XQueries and visualize the returned XML result in various ways, including raw XML, HTML and 3-D.

**Figure 6:**
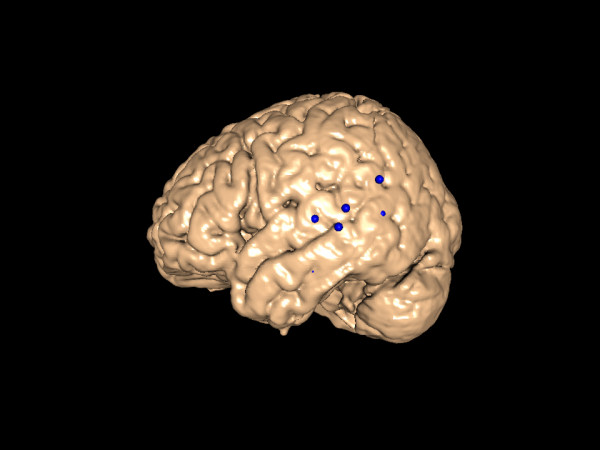
**Query Results**. Results of a query by our DXBrain application, displayed on a canonical brain. The colored spheres represent the locations of temporal lobe CSM sites in which male patients made semantic (as opposed to syntactic) language errors in response to cortical stimulation. The locations, sizes and colors of the spheres were passed to MindSeer via the information system.

When the user requests a 3-D visualization of the returned data, DXBrain reformulates the returned XML results as a MindSeer library file, and automatically displays the results on a choice of 3-D surfaces – either a canonical brain surface to which all data have been normalized, or a patient-specific cortical surface from our repository. For the canonical brain surface the system downloads the data and launches MindSeer in standalone mode through a Java Web Start link (Figure 6). The download for the canonical surface only happens the first time MindSeer is launched, so the extra speed and interactivity of standalone mode (which takes advantage of the client graphics hardware) makes this small download worth the extra time. However, when the user selects a brain from our repository, the required download for each subject would be too large and expensive. Therefore, we use the versatility of client-server mode for visualizing data on a single subject. The two different modes are transparent to the user except for the slower response time in client-server mode.

The ability to dynamically visualize query results with a single click of a web link has proved to be an invaluable tool for our collaborators, who can quickly adapt and refine queries to highlight the results in which they are interested. It has also proven to be a thorough test of MindSeer, as it has routinely and successfully been used on Mac, Linux and Windows systems with various hardware and browser configurations.

### Extending MindSeer

**Figure 7:**
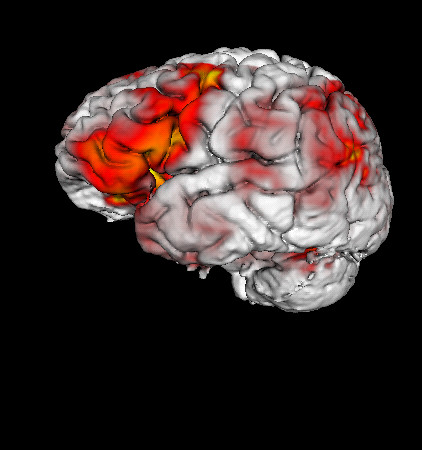
**fMRI Surface Coloring Plugin**. The plugin colors a cortical surface based on activation found in the corresponding fMRI volume dataset.

To test the extensibility of MindSeer a computer science undergraduate student, (author PL), wrote a series of plugins as a senior thesis project [41]. The goal of this work was to explore ways to highlight the cortical surface with functional data sources. The final result added the capability to color cortical surfaces with functional data like fMRI or source localized EEG (Figure 7). In order to integrate his algorithms as plugins he needed to implement two subclasses of the abstract plugin classes- a data wrapper around a volume data model to add new visualization capabilities, and a custom viewport controller. The wrapper binds his surface coloring algorithms, the volume data model and the underlying cortical surface data model. The custom controller allows the user to specify the exact surface coloring algorithm, as well as how multiple functional volumes interact.

The new data volume wrapper made it easy to leverage existing decoders for surface and volume data, as well as the corresponding data models. It also made it possible for his subsystem to handle new decoders for volumetric data without modification. Furthermore, by using the specialized GUI widgets in his controller, his subsystem was able to work in client-server mode without additional effort. The default user interface layout also made it so that his controls naturally work with the existing interface. The end result is that much of his time was spent experimenting with visualization algorithms instead of reinventing the wheel.

## Discussion

These five case studies show that MindSeer achieves the ten design goals previously described. The achievement of these goals provides a basis for future enhancements. In particular, the plugin mechanism should allow an undergraduate-level computer science or similar student to add new image modalities and visualization approaches with relative ease. For example, the ability to plot points could be extended to draw curves and to calculate areas, as in the Measure program [42].

Furthermore, there is nothing in the code that restricts MindSeer to functional brain imaging data; with proper file encoders and decoders or external file converters [43], the system should be able to display gene expression data on developmental atlases such as the mouse [44], and with new data model plugins should be able to display 3-D molecular structures [45]. Other data that can be placed in a 3 or 4D grid could be visualized using MindSeer. These include computed tomography (CT), among others. Additionally, while MindSeer's default units are in millimeters, the units can be configured to display sub-millimeter data such as μMRI, optical projection tomography (OPT) [46], episcopic fluorescence image capture (EFIC) [47,48] and confocal microscopy.

Other possible enhancements include the ability to display 4-D data via animations or a time slider in the 3-D viewport; the ability to complement the Java Web Start client with an AJAX [49] style client similar to Google Maps [50], thereby eliminating all setup requirements; the ability to click on regions of the 3-D brain and send queries to the backend databases, thereby allowing MindSeer to be a visual query interface as well as a display interface; the ability to generate 3-D reconstructions from serial sections [51]; and the ability to generate interactive anatomical scenes for anatomy education [52]. We believe that the addition of each of these features would be relatively straightforward because of MindSeer's modular and extensible architecture, all of which is open source.

## Conclusion

In this paper we have presented MindSeer, a new application for visualizing multimodality neuroimaging data that is extensible to other biomedical data as well. Although there are many 3-D visualization programs available, MindSeer combines the best of many of them while retaining portability and extensibility. In addition, MindSeer is free and open source, thereby making it a candidate for extension to the needs of specific user communities.

## Availability and Requirements

• **Project name: **MindSeer

• **Project home page: **

• **Operating system(s): **Windows, Linux, Mac

• **Programming language: **Java, Java3D

• **Other requirements: **Java 1.5+, Java3D 1.3

• **License: **GPL

• **Any restrictions to use by non-academics: **None

## Abbreviations

3-D – three-dimensional

AJAX – asynchronous JavaScript and Xml

CSM – Cortical Stimulation Mapping

ERP – Event related potential

fMRI – functional magnetic resonance imaging

GPL – GNU General Public License

GUI – graphical user interface

HTML – Hypertext Markup Language

MRI – Magnetic resonance imaging

MVC – Model-view-controller

NIFTI – Neuroimaging Informatics Technology Initiative

RMI – Remote Method Invocation

XML – Extensible Markup Language

## Competing interests

The author(s) declares that there are no competing interests. 

## Authors' contributions

EM – primary designer and implementer of MindSeer, primary author of manuscript.

AP – neuroscience user requirements, software tester, user and maintainer.

PL – fMRI surface coloring plugin.

JB – lab director, problem and use case specifications, software tester, manuscript preparation, funding support.

All authors have read and approved the final version of the manuscript.
